# Immune Activation, Proinflammatory Cytokines, and Conventional Risks for Cardiovascular Disease in HIV Patients: A Case-Control Study in Bahia, Brazil

**DOI:** 10.3389/fimmu.2018.01469

**Published:** 2018-06-26

**Authors:** Clara Brites-Alves, Estela Luz, Eduardo M. Netto, Thalis Ferreira, Ricardo Sohbie Diaz, Celia Pedroso, Kimberly Page, Carlos Brites

**Affiliations:** ^1^LAPI – Laboratório de Pesquisa em Infectologia, Complexo Hospitalar Prof. Edgard Santos, Universidade Federal da Bahia, Salvador, Brazil; ^2^Laboratório de Retrovirologia, Universidade Federal de São Paulo, São Paulo, Brazil; ^3^University of New Mexico, Albuquerque, NM, United States

**Keywords:** HIV, cytokines, cardiovascular, immune activation, dyslipidemia

## Abstract

**Background:**

Cardiovascular events (CVE) are an increasing cause of morbi-mortality for HIV patients. The antiretroviral therapy (ART), persistent immune activation, and life style are factors that can increase CVE for such patients. We performed a case-control study to evaluate the role of coinfections and immune markers associated with CVE.

**Methods:**

We included patients under ART, with undetectable plasma viral load ≥12 months. Patients presenting any condition of risk for CVE were considered cases, and those without CVE risk conditions were controls. History of viral infections (Epstein–Barr virus, hepatitis C virus, hepatitis B virus, and cytomegalovirus), exposure to antiretroviral drugs, time since HIV diagnosis/under ART, and life style (demographics, weight, smoking, alcohol, and illicit drug use) were assessed. CD4/CD8 nadir and current counts, nadir and current CD4/CD8 ratio, immune activation markers (CD4CD38HLADR, CD8CD38HLADR), and serum levels of eight cytokines [IL-2, IL-4, IL-6, IL-10, tumoral necrosis factor-alpha (TNF-α), interferon gamma, macrophage inflammatory proteins 1 alpha, and interferon-inducing protein (IP-10)] were measured.

**Results:**

Two-thirds of patients were males. Cases (*N* = 106) were older (52.8 vs 49.5 years, *p* = 0.002), had higher levels of creatinine (0.97 vs 0.87 mg/dL, *p* = 0.002) and IL-6 (0.67 vs 0.52 pg/mL, *p* = 0.04) than controls (*N* = 114). There was no difference between groups regarding frequency of CD4CD39HLADR+ or CD8CD38HLADR+ cells. We found a significant correlation (all patients) between increased frequency of CD4CD38HLADR+ cells and levels of IP-10 (*r* = 0.171, *p* = 0.02) and TNF-α (*r* = 0.187, *p* = 0.01). Levels of IL-6 (*r* = 0.235, *p* = 0.02), TNF-α (*r* = 0.267, *p* = 0.01), and IP-10 (*r* = 0.205, *p* = 0.04) were correlated with CD4CD38HLADR+ cells, in controls. Higher frequency of CD4CD38HLADR+ cells was also correlated with levels of IP-10 (*r* = 0.271, *p* = 0.04) in patients presenting with arterial hypertension. Frequency of CD4CD38HLADR+ cells was negatively correlated with levels of IL-2 (*r* = −0.639, *p* = 0.01) and IL-6 (*r* = −0.0561, *p* = 0.03) in patients with hypercholesterolemia. No association was detected between viral infections or smoking/alcohol use and immune activation markers.

**Conclusion:**

Our results indicate IL-6 levels are associated with increased CV risk. Activated CD4+ T cells were associated with increased levels of proinflammatory cytokines.

## Introduction

The life expectancy of people living with HIV (PLHIV) has substantially increased over the last decades (1–3). In Brazil, at least 30% of PLHIV are older than 50 years, with a detection rate of new HIV infections increasing fast (by 14% in recent years) in this age range (4). A recent mathematical model (ATHENA Cohort) estimates that 73% of PLHIV will be older than 50 years by 2030 (5). This trend has led to an increasing proportion of patients affected by non-communicable diseases (NCDs), in countries where antiretroviral treatment is available (5–8).

The increasing incidence of NCD among PLHIV presents emergent challenges for clinical care and public health (5, 9, 10). Currently, the proportion of deaths caused by NCD in PLHIV is higher than those related to AIDS (6, 10, 11). Increases in NCD causes of death include cancer (not related to HIV), diabetes, nephropathy, liver disturbances, and especially cardiovascular disease (CVD), comparable to seronegative individuals at similar age.

Risk of CVD, a common NCD in aging HIV patients, may be linked to viral coinfections, concomitant T-cell activation, and increased levels of proinflammatory cytokines (12–16).

Inflammatory cytokines [like tumoral necrosis factor-alpha (TNF-α), interferon gamma (IFN-γ), and IL-6] are important players in lipid metabolism. For instance, TNF-α promotes changes in lipids levels of HIV patients by affecting lipolysis, the adipose tissue lipoprotein lipase and the synthesis of hepatic fatty acids (17). Other proinflammatory cytokines can alter levels of circulating lipids. IL-4 counterbalances the proinflammatory cytokines effects, while IL-10 increases the transformation of lipoproteins into foam cells (18, 19). Although there are consistent evidences on the role of proinflammatory cytokines on lipids metabolism, the mechanism is not clear. However, increased lipids level is a typical risk factor for CVD, while chronic inflammation is considered a potential trigger for the occurrence of such health problems.

We aimed to investigate immune activation in HIV-infected patients and the relationship with cardiovascular risk factors in a well-characterized, stable population under outpatient clinic follow-up in Salvador, Brazil.

## Materials and Methods

### Study Design and Population

The AIDS clinics of Federal University of Bahia Hospital (UFBA) follow up to 3,000 HIV patients a year. All of them are regularly seen, with standardized and systematic clinical follow-up. We used a non-matched, case-control study design. Cases were defined as HIV-infected patients *recently* diagnosed *(last 2 years)* with cardiovascular (CV) comorbidity (acute myocardial infarction, coronary *arterial* disease, hypertension, stroke, diabetes, and dyslipidemia) in the past 2 years, both genders, 35 years and older, on stable antiretroviral therapy (ART), and HIV RNA plasma viral load <50 copies for at least 12 months. Controls were HIV-infected patients with no diagnosis of CV disease based on medical record review of the past 2 years, 35 years and older, on stable ART, and HIV RNA plasma viral load <50 copies for at least 12 months. Patients were compared with respect to age, sex, life style-related variables [weight, smoking, and history of illicit drug use (marijuana, cocaine, crack, and intravenous cocaine)], prevalence of syphilis, hepatitis C virus (HCV), hepatitis B virus (HBV), human T-cell lymphotropic virus (HTLV), cytomegalovirus (CMV), and Epstein–Barr virus (EBV), and differences in levels of proinflamatory cytokines/chemokines, and markers of immune activation.

Cases and controls were identified through medical record review of UFBA’s AIDS outpatient clinics. Medical releases were obtained from patients to gather all relevant information regarding non-infectious conditions, including cardiovascular, pulmonary, renal, lipids, bone abnormalities, central nervous system, and malignancies. Blood samples were collected to confirm coinfection status, and for evaluation of immune activation and inflammatory markers. We used the recommendations of the American Association of Clinical Endocrinologists and American College of Endocrinology to define dyslipidemia (20).

The *serum* levels of eight different cytokines were measured by a multiplex biometric immunoassay containing monoclonal antibody-conjugated microspheres for protein targets revealed by fluorescent staining for cytokine quantification, according to the manufacturer’s instructions (Bio-Plex Human Cytokine Assay; Bio-Rad Inc., Hercules, CA, USA). We compared levels of cytokines [IL-2, IL-4, IL-6, IL-10, IFN-γ, TNF-α, macrophage inflammatory proteins 1 (MIP-1)-α, interferon-inducing protein (IP)-10, and markers of T-cells activation (CD4, CD8, CD38, and HLADR)] for patients with and without CV comorbidity. All infections were detected by serology. *HIV, HCV, HBV, and HTLV infections were confirmed by PCR*.

Frequency of CD4CD38HLADR+ and CD8CD38HLADR+ cells were measured by flow cytometry (FacsCalibur, Becton Dickinson, *San Jose, CA*, USA). *Briefly, 20 µL of Multitest (CD3/CD4/CD8/CD38/HLA) monoclonal antibodies were added. Monoclonal antibodies were added to Trucount tubes containing reference beads (Becton Dickinson Immunocytometry Systems, San Jose, CA, USA). 50 µL of whole blood was mixed and incubated at room temperature for 20 min in dark. Red blood cells were lysed by adding 450 µL of fluorescence-activated cell sorter lysing solution (Becton Dickinson Immunocytometry Systems). The tubes were incubated at room temperature for 20 min, and samples were analyzed with the Multiset software (Becton Dickinson Immunocytometry Systems) within 6 h*.

### Statistical Analysis

Frequency distribution of the variables means and measures of dispersion (SD) for continuous variables with normal distribution were calculated. For variables with non-normal distribution, the median and interquartile range (IQR) were used. Nominal and ordinal variables were presented as proportions. Chi-square or Fisher’s exact test were used to test associations between independent variables and CV outcome. The correlation between the cytokines and frequency of activated CD4/CD8+ T cells were calculated assuming non-normal distribution using Spearman’s rho coefficient. If the distribution was not normal, the Kruskal–Wallis test was used. All tests comparing the groups were two-sided at a level of 0.05 (α = 0.05). Statistical analysis was performed by using the statistical package SPSS^®^ (version 18.0).

### Ethics Considerations

The study is part of a larger project and was approved by institutional ethics in research committee, number 1035.826, April 2015. All patients provided a written informed consent, before entering the protocol. The study was conducted according to the Helsinki’s declaration.

## Results

We included 220 patients in the study (106 cases, 114 controls). Table 1 summarizes the main characteristics of cases and controls. Cases were older, more likely to be retired, and included a higher proportion of males than controls. On the other hand, more controls reported the use of illicit drugs than cases*. We detected no case of alcohol addiction in the study sample*. However, weight, route of HIV infection, and frequency of smoking were similar for both groups. Table 2 shows the main clinical and laboratorial findings for included patients. Regarding the detected conventional risks for CV comorbidities (cases), the most prevalent was arterial hypertension (64.2%), followed by hypercholesterolemia (19.8%), hypertriglyceridemia (18.8%), and mixed dyslipidemia (17.9%). The frequency of other comorbidities (viral coinfections, syphilis, bone disturbances, and neoplasms) was similar for cases and controls. Almost all (>98%) patients had a positive serology for CMV and EBV. One patient tested positive for HTLV-1 and one for syphilis, both were cases.

**Table 1:** Demographic and laboratorial characteristics of cases and controls.

Characteristic	Cases (*N* = 106)	Controls (*N* = 114)	*p* Value
	*N* (%)/mean ± SD	*N* (%)/mean ± SD	
Age	52.5 ± 9.7	48.8 ± 8.5	0.02
**Sex**			
Male	65 (61.3)	83 (72.8)	0.04
Female	41 (38.7)	31 (27.2)	
**Education**			
<12 years	46 (44.2)	58 (40.5)	0.58
≥12 years	58 (55.8)	66 (59.5)	
Weight (kg)	74.1 ± 16.1	71.6 ± 12.6	0.2
Years on ART	12.5 ± 6.5	11.1 ± 6.6	0.09
**Smoking**			
Current	12 (11.3)	20 (17.5)	0.4
Past	25 (23.6)	26 (22.8)	
**Civil status**			
Steady partner	24 (22.9)	19 (16.8)	0.2
Single or widowed	82 (77.1)	94 (83.2)	
Retired	52 (49.1)	31 (27.2)	<0.01
HIV risk profile			0.85
Heterosexual	51 (52.6)	55 (53.9)	
MSM/bisexual/IV drugs	46 (47.4)	47 (46.1)	
History of illicit drug use	2 (1.9)	10 (9.4)	0.03
Nadir CD4 count	299 ± 238	333 ± 232	0.3
Current CD4 count	778 ± 350	750 ± 350	0.5
Current CD4/CD8 ratio	0.86 (0.45)	0.9 (0.49)	0.55
Platelet count	236,882 ± 73,933	217,177 ± 81,012	0.09
Creatinine (mg/dL)	0.97 ± 0.27	0.87 ± 0.16	0.002
AST (mg/dL)	32.2 ± 21.7	31.6 ± 28.1	0.8
ALT (mg/dL)	36.5 ± 34.9	35.1 ± 33.9	0.8

*AST, aspartate aminotransferase; ALT, alanine aminotransferase; ART, antiretroviral therapy*.

**Table 2:** Frequency of diagnosed cardiovascular diseases, coinfections, and other comorbidities in cases and controls.

Comorbidity	Cases	Controls	Total	*p* Value
	*N* (%)	*N* (%)	*N* (%)	
Hypertension	68 (64.2)	–	68 (30.1)	–
Myocardial infarction	1 (0.9)	–	1 (0.5)	–
Stroke	3 (2.7)	–	3 (1.3)	–
Diabetes	13 (12.3)	–	13 (5.9)	–
Coronary disease	2 (1.8)	–	2 (0.9)	–
Dyslipidemia	19 (17.9)	–	19 (8.6)	–
Renal dysfunction	3 (2.7)	2	5 (2.3)	0.8
AIDS-related neoplasm	0	0	0	–
Non-AIDS neoplasm	3 (2.7)	0	3 (1.4)	0.1
Osteopenia	2 (1.8)	3 (2.6)	5 (2.3)	0.5
Osteoporosis	5 (4.5)	1 (0.9)	6 (2.7)	0.09
Hepatitis C virus	6 (5.4)	7 (6.1)	13 (5.9)	0.5
Hepatitis B virus	0	3 (2.6)	3 (1.4)	0.1

The only difference between groups was detected in levels of IL-6, which were significantly higher in cases than in controls (Table 3). The frequency of CD4CD38HLDR+ or CD8CD38HLADR+ cells was similar for cases and controls.

**Table 3:** Frequency of CD4CD38HLADR+, CD8CD38HLADR+ cells (%), and serum levels of eight cytokines (pg/mL) in cases and controls.

	Cases		Controls		
Cytokine/activated T cells	Median	Interquartile range (IQR)	Median	IQR	*p* Value
IL2	0.93	(0.23–0.73)	0.93	(0.71–1.48)	0.81
IL4	12.94	(2.06–45.44)	5.79	(1.38–37.62)	0.15
IL6	0.67	(0.43–1.12)	0.52	(0.37–0.87)	0.04
IL10	0.58	(0.25–1.45)	0.56	(0.25–1.03)	0.69
Tumoral necrosis factor-alpha	7.81	(2.94–11.24)	7.26	(1.68–11.24)	0.55
Interferon-gamma	2.62	(1.49–8.40)	3.02	(1.73–8.64)	0.48
Interferon-inducing protein-10	201.6	(136.5–284.9)	202.9	(132.3–307.2)	0.74
Macrophage inflammatory proteins 1 alpha	5.34	(2.43–17.81)	5.41	(2.22–14.81)	0.61
CD4CD38HLADR+	4.23	(2.66–7.08)	4.21	(2.55–7.46)	0.92
CD8CD38HLADR+	4.56	(2.93–9.01)	4.78	(3.37–8.35)	0.71

We detected a significant correlation between frequency of CD4CD38HLDR+ cells and levels of IP-10 (*r* = 0.205, *p* = 0.04), TNF-α (*r* = 0.267, *p* = 0.01), and IL-6 (*r* = 0.235, *p* = 0.02) in control group (Table 4). When we looked at all patients, frequency of CD4CD38HLDR+ cells was significantly correlated with levels of IP-10 (*r* = 0.171, *p* = 0.02) and TNF-α (*r* = 0.187, *p* = 0.01), but not with IL-6 levels. We did not detect any correlation between frequency of CD8CDE38HLADR+ cells and cytokines’ levels.

**Table 4:** Correlations (Spearman’s rho coefficient) between frequency of CD4CD38HLADR+/CD8CD38HLADR+ cells and serum levels of eight cytokines for cases and controls.

Cytokines	CD4+CD38+HLADR+		CD4+CD38+HLADR+		CD8+CD38+HLADR+	CD8+CD38+HLADR+
	Cases	Controls	Cases	Controls	All patients	All patients
IL-2	−0.075	0.056	−0.491	0.049	0.008	−0.028
IL-4	−0.081	−0.047	−0.096	0.118	−0.062	0.007
IL-6	−0.047	0.235*	−0.041	0.127	0.104	0.039
IL-10	−0.045	−0.045	0.016	−0.074	−0.039	−0.017
Tumoral necrosis factor-alpha	0.63	0.267*	0.068	0.199	0.187*	0.151
Interferon gamma	0.019	0.015	−0.095	0.100	0.06	−0.09
Interferon-inducing protein-10	0.141	0.205*	−0.069	0.134	0.171*	0.054
Macrophage inflammatory proteins 1 alpha	−0.18	−0.006	0.144	−0.006	0.008	0.039

*p < 0.05 for the correlation between frequency of activated T cells and cytokines’ levels.

In patients presenting with arterial hypertension, there was a significant correlation between frequency of CD4CD38HLADR+ cells and IP-10 (*r* = 0.271, *p* = 0.04). Among patients presenting with hypercholesterolemia, the frequency of CD4CD38HLDR+ cells were negatively correlated to IL-6 (*r* = −0.561, *p* = 0.03) and to IL-2 levels (*r* = −0.630, *p* = 0.01). However, when we grouped patients with normal serum lipids, we detected a significant correlation between CD4CD38HLDR+ cells and levels of IP-10 (*r* = 0.221, *p* = 0.01), MIP-1-α (*r* = 0.258, *p* = 0.04), and TNF-α (*r* = 0.242, *p* = 0.005). Table 3 details the differences between serum levels of different cytokines for cases and controls, and Table 4 shows the detected correlations between T-cells activation markers and cytokine levels, for cases and controls. Table 5 summarizes the correlations observed between cytokines, activated T cells, and hypertension/hypercholesterolemia.

**Table 5:** Correlations (Spearman’s rho coefficient) observed between T cells activation markers and cytokines levels, according to the presence of traditional cardiovascular risk in HIV patients on stable therapy.

Cytokines	CD4CD38HLADR+		CD8CD38HLADR+	
	Cholesterol	Hypertension	Cholesterol	Hypertension
IL-2	−0.630*	−0.008	−0.227	−0.026
IL-4	0.066	−0.002	0.246	0.030
IL-6	−0.561*	0.117	−0.196	0.110
IL-10	−0.289	0.033	−0.011	0.073
Tumoral necrosis factor-alpha	0.095	0.158	0.502	0.110
Interferon gamma	−0.404	0.151	−0.241	0.016
Interferon-inducing protein-10	−0.164	0.271*	−0.323	0.130
Macrophage inflammatory proteins 1 alpha	−0.221	−0.008	−0.095	0.215

*p < 0.05 for the correlation between frequency of activated T cells and cytokines’ levels.

*Over 50 different antiretroviral regimens (ART) were in use by patients at study entry: the most frequent regimen (33%) was efavirenz-based ART, followed by protease/inhibitors-based regimens (25%), and nevirapine-based ones (16%)*.

## Discussion

We detected a significant correlation between T-cells activation markers, levels of cytokines and presence of specific CVD risks. The only difference between cytokines levels for cases and controls was detected for IL-6 levels, which were significantly higher in cases than in controls. However, we detected significant correlations between frequency of cellular immune activation markers and proinflammatory cytokines (IP-10 and TNF-α) for the entire population of study. Moreover, we found a significant correlation between activated CD4+ T cells and IP-10, in patients presenting with arterial hypertension. However, levels of IL-2 and IL-6 were negatively correlated with activated CD4+ T cells in patients with hypercholesterolemia.

Higher levels of IL-6 are associated with increased CVD risk, and in SMART study, increased IL-6 levels were highly predictive of death or incident CV events (21). In our study, IL-6 levels were significantly higher in cases than in controls. In addition, when we looked at all patients, we detected a significant correlation between frequency of CD4CD38HLADR+ cells and levels of TNF-α and IP-10. These cytokines are involved in activation of several cell types, chemotaxis and inflammatory response, and activated CD4+ T cells are likely to increase their production (22). In a recent work involving 252 HIV patients Mooney et al. detected that, despite viral suppression and immunological stability, levels of TNF-α and other inflammatory biomarkers are persistently elevated in a large proportion of HIV patients, and this fact is associated with increased cardiovascular risk, as measured by Framingham and Veterans Aging Cohort Study scores (23).

Tumoral necrosis factor-alpha and IP-10 were consistently correlated with frequency of activated CD4+ T cells for controls and pooled groups, but for IL-6, this relationship was seen only in control group. However, Il-2 and IL-6 were negatively correlated with activated CD4+ T cells in patients presenting with hypercholesterolemia. In addition, among patients without dyslipidemia, levels of IP-10 and TNF-α were significantly correlated with CD4CD38HLDR+ cells, while levels of MIP-1-α were correlated with CD8CD38HLDR+ cells in patients with normal lipids’ levels. These findings suggest that IP-10 and TNF-α are involved in lipids’ regulation and their levels are associated with cellular immune activation.

Tumoral necrosis factor-alpha can affect the metabolism of lipids, by modifying important functions like lipolysis, adipose tissue lipoprotein lipase, and synthesis of fatty acids by the liver (17, 19, 23). In addition, IL-6 and TNF-α stimulates the hepatic lipogenesis and were also associated with lipodystrophy in HIV patients (21, 24, 25). The detected correlation between CD4CD38HLDR+ cells and IP-10 suggests that this cytokine is involved in persistent immune activation (PIA) and inflammation. In addition, IP-10 levels were significantly correlated with CD4CD38HLDR+ cells in patients with arterial hypertension, a well-known risk factor for cardiovascular events*. The detected correlation between CD8CD38HLDR*+ *and MIP-1-α reinforces the link between T cell activation and inflammation, as events that can drive increased CVD risk*.

Interferon-inducing protein-10 is produced by several cell types (including monocytes, endothelial cells, and fibroblasts) in response to IFN-γ. IP-10 promotes chemo attraction (for monocytes/macrophages, T cells, NK cells, and dendritic cells), T cell adhesion to endothelial cells, antitumor activity, and inhibition of bone marrow colony formation and angiogenesis (26). Levels of IP-10 are usually elevated in patients with chronic untreated HCV infection (27). *Some previous reports indicate that IP-10 either directly or indirectly can influence hepatic lipogenesis and contributes to liver steatosis and inflammation* (28). Our results show increased levels of IP-10 in patients with higher frequency of CD4CD38HLDR+ cells, as well as in those presenting with arterial hypertension. These findings suggest IP-10 plays a role as a marker of immune activation and, perhaps, of arterial hypertension, for HIV-infected patients. TNF-α is produced by macrophages and many other cells, including T lymphocytes. It primary role is regulation of immune cells, but it is also involved in inflammation and many other activities, including regulation of lipids metabolism (21, 24, 25). The consistent correlations seen in our study between CD4CD38HLDR+ and CD8CD38HLDR+ cells and TNF-α suggest that immune activation is closely related with inflammation and CVD risks in HIV patients on stable antiretroviral treatment.

Persistent immune activation is often associated with microbial translocation, ongoing HIV replication, and viral coinfections (14, 15). However, in many of available reports the studied population was heterogeneous and included patients with detectable viremia or clinical manifestations of disease. In our study, we included only patients on stable therapy, with long-term HIV suppression, and with no current HIV-related events. We did not see any association between prevalent viral coinfections and signs of immune activation or inflammation. However, activated CD4CD38HLDR+ cells were significantly correlated with increased levels of IP-10 and TNF-α when we looked at all patients. IP-10 was also associated with hypertension, and controls showed a significant correlation between frequency of CD4CD38HLDR+ and levels of IL-6, TNF-α, and IP-10.

On the other hand, there was a consistent correlation between hypertension and frequency of CD4CD38HLDR+ cells. T cells activation was also correlated with increased production of proinflammatory cytokines, like IP-10, IL-6, and TNF-α. Our findings suggest that in treated patients activated T cells are involved in inflammation and can affect CV risk in HIV-infected patients.

The reasons for T cells activation were not clear in our study. Chronic CMV and EBV infections were seen in over 98% of our patients and would not explain the observed T cells activation. Other chronic viral infections were infrequent in the studied groups and were not associated with immune activation nor with increased levels of proinflammatory cytokines. Other causes, like microbial translocation, were not investigated, and would be *less frequent* in patients with high CD4+ cells count and suppressed viremia.

IL-6 is an already established marker for CV risk in HIV patients. Our results showed higher levels of IL-6 among cases, and a significant association between TNF-α and IP-10 for all patients. This indicates that cellular immune activation can trigger the observed increase in proinflammatory cytokines and, in consequence, increase the risk of NCD in HIV patients on stable therapy. *A long-term follow-up of patients would provide an opportunity to evaluate the NCD outcomes among those presenting signs of immune activation and inflammation*.

The case-control design of this study involves retrospective analyses of exposures in association with the outcome, limiting conclusions on causality with the observed events. However, both cases and controls are drawn from the same clinical study base, which was mostly homogeneous, on stable therapy and long-term viral suppression. These characteristics made possible to exclude potential confounding factors, like ongoing viremia, or AIDS-related comorbidities. The low prevalence of dyslipidemia in our study’s population probably affected the power to detect associations between cytokines’ levels and lipids. *In addition, because of the great variety of ART regimens used by patients, it was not possible to assess the potential effect of distinct antiretroviral drugs on lipids and immune activation*.

Understanding chronic micro-inflammation is very important in HIV disease. We detected a significant association between inflammatory markers, cytokines, and hypertension. Taken together, our findings indicate that activated CD4+ T cells are linked to immune dysregulation, with increased production of proinflammatory cytokines even in patients with prolonged viral suppression. These effects can interfere on lipids metabolism and increase the risk of development of CVD in HIV-treated population.

## Ethics Statement

The Project was approved by the Ethics Committee of Maternidade Climério de Oliveira (UFBA), number 1.035.826, on April 26, 2015.

## Author Contributions

CB-A, EL, EN, KP, RD, and CB have designed the study, supervised data collection and statistical analysis. TF and CP were responsible for collecting and testing samples. CB-A, KP, RD, and CB were responsible for writing the paper.

## Conflict of Interest Statement

The authors declare that the research was conducted in the absence of any commercial or financial relationships that could be construed as a potential conflict of interest.
